# Retrograde Cerebral Air Embolism in a Patient with Intestinal Necrosis: A Case Report

**DOI:** 10.4274/balkanmedj.2016.0292

**Published:** 2017-08-04

**Authors:** Onur Taydaş, Mehmet Ruhi Onur, Erhan Akpınar

**Affiliations:** 1 Department of Radiology, Hacettepe University School of Medicine, Ankara, Turkey

**Keywords:** Mesenteric ischaemia, air embolism, Necrosis, Multidetector computed tomography

## Abstract

**Background:**

Cerebral venous air embolism is a severe clinical condition related to an unfavourable outcome in patients with neurological impairment. Cerebral venous air embolism may occur secondarily to arterial or venous interventions. A rare mechanism of cerebral venous air embolism is retrograde embolism, which is characterized by gas flow in a direction that is opposite to that of the normal blood flow.

**Case Report:**

A 69-year-old female was admitted to our hospital with shortness of breath and abdominal pain. Abdominal computed tomography revealed intramural gas in the bowel and free gas in the mesenteric veins and portal vein. Cranial computed tomography, which was performed due to impaired consciousness, demonstrated cerebral air embolism with the appearance of a gyriform pattern. A bedside echocardiography and chest computed tomography revealed no evidence of right-to-left shunt.

**Conclusion:**

Cerebral venous air embolism may occur after pneumatosis intestinalis by a retrograde flow of air from the mesenteric veins and portal vein. Low cardiac output and supine position are contributing factors for a retrograde flow of air bubbles into the venous circulation of the brain.

Cerebral venous air embolism (CVAE) is a rare but critical situation. It is generally caused by arterial and venous interventions. Presence of CVAE has been defined as as having a poor prognosis in patients with impaired clinical status (1). Hyperbaric oxygen therapy can be used to overcome neurological complications of CVAE (1). 

Pneumatosis intestinalis (PI) is characterized by the accumulation of air in the bowel wall. This phenomenon may be observed in many different clinical conditions, such as intestinal ischaemia and/or necrosis, chronic obstructive pulmonary disease and steroid use. Presence of air in the main portal vein and its branches in the setting of PI is a sign of bowel necrosis (2).

To the best of our knowledge, the occurrence of CVAE in the setting of intestinal necrosis has never been reported in the literature. In this presentation, we aim to present computed tomography (CT) features of CVAE resulting from the retrograde flow of air from the mesenteric veins and the portal vein.

## CASE PRESENTATION

**Figure 1:**
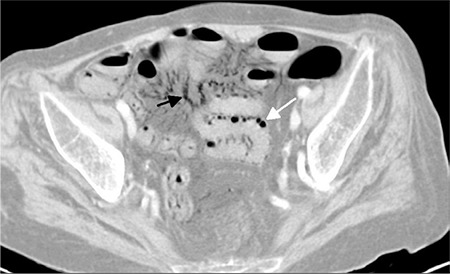
Contrast-enhanced abdominal computed tomography shows pneumatosis intestinalis (white arrow) and air in mesenteric vessels (black arrow)

**Figure 2:**
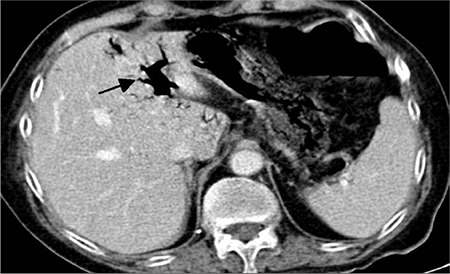
Contrast-enhanced abdominal computed tomography demonstrates air in portal vein (black arrow).

**Figure 3:**
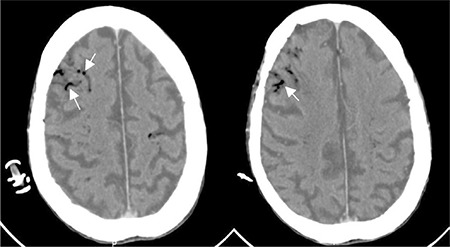
Unenhanced head computed tomography reveals cerebral air (white arrows) distributed in gyriform pattern in both cerebral hemispheres.

A 69-year-old female was admitted to our hospital with shortness of breath and abdominal pain. She had a history of chronic kidney disease and haemodialysis with a tunnelled central venous catheter (CVC) for 18 years. Her laboratory tests revealed impaired kidney functions. No significant pulmonary abnormality was detected on the chest radiography and CT (Emotion Duo; Siemens, Erlangen, Germany). CT of the abdomen revealed intramural gas in the bowel wall with accompanying free gas in the mesenteric veins and portal vein branches (Figure 1,Figure 2). A head CT of the patient performed due to impaired consciousness demonstrated free gas in the brain distributed in the gyriform pattern, which was attributed to CVAE (Figure 3). A bedside echocardiography (Acuson; Siemens, Erlangen, Germany) of the patient revealed low cardiac output without evidence of right-to-left cardiac shunt. There was no septal defect or vascular abnormality on the chest CT suggesting right-to-left shunt. No invasive vascular procedure such as CVC insertion was performed. During follow-up in the intensive care unit, the patient became hypotensive and died due to uncontrolled haemodynamic impairment. Written informed consent was obtained from the patient's family who participated in this study.

## DISCUSSION

Cerebral air embolism is a life-threatening condition with a mortality rate of approximately 25% (3). CVAE mostly occurs as a result of insertion, maintenance or removal of a CVC (2). Clinical outcome of CVAE ranges between focal cerebral symptoms, encephalopathy, seizure and coma (3). CVAE can result from two mechanisms: paradoxical embolism and retrograde venous air embolism. Paradoxical embolism is characterized by the air passage from the venous system to arterial circulation through the cardiac or pulmonary right-to-left shunts, such as patent foramen ovale and pulmonary arteriovenous malformations, respectively. Paradoxical embolism can also occur in the absence of right-to-left shunts due to certain factors, such as a large volume of intravascular air, use of vasodilators or anaesthetic agents, which facilitate air passage through pulmonary capillary beds by compromising the ability of pulmonary capillaries to filter out gas emboli (3,4,5). In the retrograde mechanism, air in the central venous circulation reaches the cerebral vasculature by moving in the opposite direction of the venous blood flow (5,6). Experimental studies have revealed that retrograde air emboli can occur in venous vasculature in the setting of certain circumstances, such as putting the patient in a supine position or at least at an angle of 45 °C to the horizontal plane, venous valve insufficiency, and increased venous pressure in the right cavities of the heart (7,8). In our patient, CVAE may have resulted from the retrograde flow of air from the mesenteric veins and portal vein followed by the passage of air through the right cardiac chambers and cerebral venous vasculature. Absence of right-to-left cardiac shunt on the echocardiography and pulmonary arteriovenous malformation on the chest CT decreased the likelihood of paradoxical embolism in this case. CVC, as a potential source of air embolism, was not inserted until the CVAE was detected. Air in the mesenteric and portal veins secondary to PI was the only detected source of the CVAE in our patient. 

PI may result the dissection of gas into the bowel wall due to increased pressure from the bowel lumen or the entrance of gas-forming bacilli in the bowel wall (2). Bowel necrosis, which can occur in the setting of PI, manifests with free gas in the lumen of the mesenteric and portal veins (9). The low cardiac output and supine position of this case facilitated a retrograde flow of air bubbles through the portal vein and central venous circulation. Air in the portal vein cannot enter the hepatic veins and inferior vena cava. We suggest that mesenteric and portal venous air in our patient passed to systemic venous circulation through portosystemic shunts such as the rectal, retroperitoneal or paraumbilical veins. Although these portosystemic shunt pathways allow blood flow between the portal venous system and the systemic venous circulation in the setting of severe portal hypertension, retrograde air flow does not require a pressure gradient to traverse portosystemic shunts. To the best of our knowledge, there is only one report in literature that presents CVAE resulting from mesenteric infarction (10). 

CVAE may manifest with various imaging appearances on CT. Cavernous sinuses are most often involved in retrograde air embolism (4). Cheng et al. (6) described the patterns of CVAE according to the distribution of air in the brain as gyriform air, venous sinus air bubbles and parenchymal/subarachnoid space air bubbles. In our case, CVAE manifested with a gyriform pattern on CT and the patient died shortly after detection of CVAE.

In conclusion, CVAE can occur secondary to bowel necrosis and the retrograde air flow from the portal venous system to the systemic and cerebral venous circulation. CVAE should be kept in mind in the differential diagnosis of patients with intestinal ischaemia and neurological impairment.
